# Affordable and equitable access to subsidised outpatient medicines? Analysis of co-payments under the Additional Drug Package in Kyrgyzstan

**DOI:** 10.1186/s12939-019-0990-6

**Published:** 2019-06-13

**Authors:** Sabine Vogler, Peter Schneider, Guillaume Dedet, Hanne Bak Pedersen

**Affiliations:** 1 WHO Collaborating Centre for Pharmaceutical Pricing and Reimbursement Policies, Pharmacoeconomics Department, Gesundheit Österreich GmbH (GÖG / Austrian Public Health Institute), Vienna, Austria; 2 Organisation for Economic Co-operation and Development (OECD), Paris, France; 3 World Health Organization, Regional Office for Europe, Copenhagen, Denmark

**Keywords:** Co-payment, Out-of-pocket payment, Access to medicines, affordability, equity, pharmaceutical policy, Pricing, Price regulation, Transparency, Evaluation

## Abstract

**Background:**

Out-of-pocket (OOP) payments can constitute a major barrier for affordable and equitable access to essential medicines. Household surveys in Kyrgyzstan pointed to a perceived growth in OOP payments for outpatient medicines, including those covered by the benefits package scheme (the Additional Drug Package, ADP). The study aimed to explore the extent of co-payments for ADP-listed medicines and to explain the reasons for developments.

**Methods:**

A descriptive statistical analysis was performed on prices and volumes of prescribed ADP-listed medicines dispensed in pharmacies during 2013–2015 (1,041,777 prescriptions claimed, data provided by the Mandatory Health Insurance Fund). Additionally, data on the value and volume of imported medicines in 2013–2015 (obtained from the National Medicines Regulatory Agency) were analysed.

**Results:**

In 2013–2015, co-payments for medicines dispensed under the ADP grew, on average, by 22.8%. Co-payments for ADP-listed medicines amounted to around 50% of a reimbursed baseline price, but as pharmacy retail prices were not regulated, co-payments tended to be higher in practice. The increase in co-payments coincided with a reduction in the number of prescriptions dispensed (by 14%) and an increase in average amounts reimbursed per prescription in nearly all therapeutic groups (by 22%) in the study period. While the decrease in prescriptions suggests possible underuse, as patients might forego filling prescriptions due to financial restraints, the growth in average amounts reimbursed could be an indication of inefficiencies in public funding. Variation between the regions suggests regional inequity. Devaluation of the national currency was observed, and the value of imported medicines increased by nearly 20%, whereas volumes of imports remained at around the same level in 2013–2015. Thus, patients and public procurers had to pay more for the same amount of medicines.

**Conclusions:**

The findings suggest an increase in pharmacy retail prices as the major driver for higher co-payments. The national currency devaluation contributed to the price increases, and the absence of medicine price regulation aggravated the effects of the depreciation. It is recommended that Kyrgyzstan should introduce medicine price regulation and exemptions for low-income people from co-payments to ensure a more affordable and equitable access to medicines.

**Electronic supplementary material:**

The online version of this article (10.1186/s12939-019-0990-6) contains supplementary material, which is available to authorized users.

## Background

After the collapse of the Soviet Union, several of the transition countries in the region moved from the Semashko health care model to a social health insurance system [1–3]. Kyrgyzstan introduced the Mandatory Health Insurance Fund (MHIF) in 1997 [4]. Since its independence in 1991, the country has continued to implement health care reforms, which have contributed to important progress: several evaluations have pointed out improvements in ensuring a more affordable access to health services [5–7].

Nevertheless, major concerns with regard to equity and accessibility remain [8]. First, since a high proportion of the population lives in rural Kyrgyzstan, ensuring access to health care– including medicines – in remote areas is a challenge [9, 10]. Second, out-of-pocket (OOP) payments for health services have been steadily increasing in the new millennium, with considerable growth in the financial burden after 2009, in particular for the poorest population groups and in the two largest cities – Bishkek and Osh [11, 12]. Findings from household surveys for 2006–2014 suggest that OOP payments for outpatient medicines, representing more than 60% of total OOP spending, were a major driver for increases in total OOP spending in Kyrgyzstan [11, 12]. On average, the Kyrgyz population saw a 4.6-fold increase in their OOP expenditure on outpatient medicines between 2006 and 2014 (a 2.8-fold increase for prescribed medicines and a 7.6-fold increase for non-prescribed medicines) [11].

The indications of rising private expenses for outpatient medicines in Kyrgyzstan suggested a need for further research into patients’ payments focused on medicines, including prescribed outpatient medicines that are included in the benefits package scheme and thus partially funded by the MHIF. Against this backdrop, the aim of the study is to explore the extent of co-payments for outpatient reimbursed medicines and to explain the reasons for evolutions, such as increases. A secondary objective of the research is to apply a different methodology: while previous health financing studies on Kyrgyzstan were based on household survey data, this research employs reimbursement claims data.

OOP payments can constitute a major barrier for affordable access to health care, including essential medicines [5, 8, 13]. High OOP payments have been reported from many countries around the world [14–16], including Central Asian countries [8, 17–21]. Catastrophic OOP payments are defined cases in which households spend more than 40% of their capacity to pay for health care, and these have been shown to give rise to difficult situations: they can pressure private households to borrow and sell assets to finance health care, and can thus cause indebtedness and lead to poverty [22–28]. In Kyrgyzstan, 12.8% of households experienced catastrophic spending on health in 2014. The proportion of households with catastrophic OOP payments had decreased substantially between 2000 and 2003, and its incidence held steady at about 10% between 2003 and 2009. Between 2009 and 2014, however, the trend reversed and the proportion increased considerably [12].

OOP payments can result from both formal and informal patient payments. Although definitions vary, the most common informal payments are defined as direct contributions made in addition to any contribution determined by the terms of entitlement, in cash or in kind, by patients or others acting on their behalf, to health care providers for services to which the patients are entitled [29, 30]. In Kyrgyzstan, informal payments – particularly for medicines, medical supplies and food – that had decreased between 2001 and 2006 [31], but started to rise again after 2006 [32].

Formal OOP payments for health services, including medicines, comprise both full patient expenses for health care whose provision is not at all covered by a third party payer (e.g. a public payer such as social health insurance) and any form of co-payments. The latter describe patients’ financial contributions (cost-sharing) to health services, including medicines, that are funded by a third-party payer; examples are fixed co-payments such as prescription fees, percentage co-payments and upfront payments through deductibles [33].

Studies analysing the impact of co-payments for medicines included in outpatient benefits package schemes have shown the effect of reductions in public pharmaceutical expenditure and also, in some but not all cases, reductions in medicine use [34–43]. In this respect, concerns have been expressed that the introduction of, or increases in, co-payments could negatively affect medication adherence since patients might decide to forego filling prescriptions for financial reasons. Existing evidence tends to confirm a negative association between (increased) co-payments and medicine use and adherence [44–50]. This adds to evidence that the introduction of coverage through a public benefits scheme as well as co-payment assistance such as reductions of and exemptions from OOP spending contributed to earlier filling of prescriptions and likely increased access and adherence [51–53]. While informal payments in health care have been investigated for several former Soviet Union countries, there is a paucity of evidence related to official co-payments for medicines included in the outpatient benefits package schemes in these countries. Existing household surveys [11, 12] identified the need for further research into the accessibility and affordability of outpatient medicines that are partially covered by the MHIF in Kyrgyzstan. This research gap is addressed in this study.

Anecdotal evidence (e.g. perceptions of patients and other stakeholders) also pointed to increases in co-payments for ADP-listed medicines, and it was suggested that high and increasing medicine prices could be accountable for these developments [54]. Since the Russian economy has been in crisis since 2014, resulting in depreciation of the Russian rouble against the US dollar – alongside depreciation of the Kyrgyz som, given Kyrgyzstan’s strong economic links to the Russian Federation – the hypothesis that increased medicine prices might be attributable to currency depreciation is also investigated in this research.

### Pharmaceutical policy framework in Kyrgyzstan

The Kyrgyz health care system has been subject to several reforms. The first was the Manas reform (1996–2006), which established the MHIF [4]. The subsequent Manas Taalimi reform (2006–2010) prioritised reduction of the financial burden on the population, along with improvements in effectiveness of health services delivery and of the quality of health care [55]. The Den Sooluk programme has been in place since 2012. This defined, among others, individual service delivery and quality of health care, health financing, access to medicines, laboratory services and aid effectiveness for universal health coverage as priority topics for action [56].

Mandatory health insurance is organised as a single payer system, with the MHIF covering 75% of the population [57]. Public coverage of medicines is provided through two schemes: the State Guaranteed Benefit Programme (SGBP) and the Additional Drug Package (ADP).

While the ‘basic benefit package’ SGBP covers various health services such as primary and secondary care and is aimed at both outpatient and inpatient sectors, the ADP is a complementary benefits scheme targeting solely medicines in the outpatient sector [4, 12, 58]. In 2015, the ADP list contained 58 medicines, listed as international non-proprietary names (INNs), and two medical devices. In the period 2013–2015, 17 medicines were delisted from the ADP. Some medicines delisted had never been included in the WHO Model List of Essential Medicines [54]. Table 1 provides a comparative overview of the two packages.

**Table 1:** Characteristics of the benefit schemes State Guaranteed Benefit Programme and Additional Drug Package in Kyrgyzstan

Benefit programme	SGBP	ADP
Full name	State Guaranteed Benefit Programme	Additional Drug Package
Year of introduction	2001, first on a pilot basis, then rolled out nationwide	2001, first on a pilot basis, then rolled out nationwide
Objectives	To improve access to defined health care services for vulnerable population groups and to increase the efficiency of health services	To improve affordability and accessibility of medicines by limiting the financial burden on households and to encourage more rational prescribing and use of medicines
Services covered	Primary, secondary and tertiary care; medicines for few defined diseases (see below)	Only medicines
Sectors covered	Outpatient and inpatient sectors	Outpatient sector
Eligibility	Any person, regardless of insurance status, with a defined eligible disease	Only patients insured by the Mandatory Health Insurance Fund (MHIF): prescriptions to be filled in community pharmacies in a contractual relationship with the MHIF
Medicines included	Coverage of medicines for defined diseases, including bronchial asthma, cancer in the terminal phase, mental disorders (schizophrenia and affective disorders) and epilepsy	Focus is on medicines for non-communicable diseases:58 international non-proprietary names (INN) of medicines and two medical devices in 2015
Co-payment	0% in principle, but some co-payments in reality as prices are not regulated	50% of the calculated tariff, but as prices are not regulated, practice rarely corresponds to 50% of the price paid by patients

Overall, public funding for both benefit package schemes for medicines is very limited: in 2017, it amounted to 1.7% of public spending on health [12].

Under the SGBP, medicines for defined conditions should be dispensed free of charge but in reality the coverage rate is around 80–90% of the retail price. In 2015, 87.9% of the costs of medicines under the SGBP was covered [59].

For ADP-listed medicines, eligible patients (i.e. the 75% of the Kyrgyz population enrolled with the MHIF) have to pay the difference between the ‘baseline price’ (the determined reimbursement tariff covered by the MHIF) and the pharmacy retail price. Medicine prices are not regulated in Kyrgyzstan, and industry and supply chain actors, including community pharmacies, are free to set and change medicine prices in accordance with their business priorities [58]. As a result, pharmacy retail prices (i.e. net prices, since essential medicines are exempt from value added tax) differ between dispensaries [57]. Medicine price information is not publicly available in Kyrgyzstan, and even the MHIF learns about the pharmacy retail prices on an ex post basis when pharmacists indicate them on the reimbursement claims. Since medicine prices are not known, the MHIF collects price data from some large wholesalers to calculate the baseline prices (participation of wholesale companies is voluntary). From the price data, the MHIF excludes the three highest and the three lowest and then calculates the average of the remaining prices to define the baseline price. The reimbursed amount (representing 50% of the baseline price) is defined using this baseline. In addition, the MHIF applies two different multipliers (one for pharmacies in urban areas; one for those in remote rural areas), resulting in two different reimbursement values (differences of around 9%) [54]. Patient co-payments are intended to amount to 50% of the pharmacy retail prices but, due to the uncertainties in the data sources for calculating baseline prices, actual co-payments for ADP-listed medicines can differ and be higher in practice. Further co-payments for medicines, such as a prescription fee or a deductible, are not applied in Kyrgyzstan, and no exemptions or reductions for the (percentage) co-payments are in place [60].

In recent years, efforts were made in Kyrgyzstan to regulate prices to make medicines more affordable. In August 2017, three strategic laws on regulating medicines and health technologies entered into force which provided a legal framework to regulate prices of medicines and medical devices in Kyrgyzstan [61]. In 2018 and 2019, the process of implementing medicine price regulation through bylaws, decrees and methodologies was ongoing.

Under the ADP, every family group medical practice may prescribe medicines up to a limit of 50.00 Kyrgyz som (0.77 United States dollars, calculated at the 2015 average exchange rate) per registered patient within 1 year. Once the prescription ceiling is reached, doctors are no longer allowed to prescribe ADP-listed medicines at the expense of the MHIF for the remainder of the year [54].

Prescription by INN is mandatory, and generic substitution is possible but not obligatory. Even though the market is mainly generic (originator medicines only account for 3% of the Kyrgyz pharmaceutical market), increasing the use of generic medicines is one of the policy aims stipulated in the National Drug Policy [57].

## Methods

### Scope of analysis

The study investigated co-payments of subsidised outpatient medicines in Kyrgyzstan (i.e. those included in the ADP list). This group of medicines was selected as representative of those that satisfy the priority health care needs of the Kyrgyz population (most of the medicines under the ADP are included in the WHO Model List of Essential Medicines; all are included in the country’s essential medicines list, since that is a prerequisite for eligibility for inclusion in the ADP list [54]).

Medicines in the hospital sector are not in the scope of the study since patient payments for medicines (both OOP payments for unfunded medicines and co-payments for subsidised medicines) had been identified as an issue for outpatient medicines.

The research was performed countrywide, with a view to identifying possible differences between the Kyrgyz regions.

When requesting primary reimbursement claims data from the MHIF, the authors had to be restrictive: extracting data for only few years was very challenging for the MHIF, considering the lack of human resources at the institution. The observation period 2013–2015 was chosen because of indications in other pieces of research that co-payments for ADP-listed medicines had increased in recent years.

The core variable studied was co-payments: average co-payments per prescription and average real co-payments as a share of the pharmacy retail price (in contrast to the theoretical share of 50%). In addition, the evolution of other relevant factors such as volume, MHIF spending and prices was investigated with regard to whether any of these could serve as possible explanations for the evolution of co-payments for medicines.

Furthermore, value and volume data of imported medicines were studied. The focus on imported medicines was justified by the fact that 97.4% of the medicines in the Kyrgyz market were imported, and only 2.6% were locally produced (data as of 2014) [57].

### Data sources

The primary source was a dataset requested and obtained from the MHIF on medicines that were reimbursed under the ADP scheme in the period 2013–2015. The dataset (*n* = 1,041,777 prescriptions claimed during 2013–2015) included the information on the price at which the medicine was sold in pharmacies (i.e. the price paid by the patient), on volumes (prescriptions) and on MHIF expenditure (i.e. reimbursement amounts) for each medicine (and medical device) prescribed and dispensed under the ADP. Furthermore, the regions in which the medicines had been dispensed were indicated. Data were provided in Russian.

In addition, data on the value and volume of imported medicines in the period 2013–2015 were requested and obtained from the National Medicines Regulatory Agency. The dataset (in Russian) contained information on the total amount (in value and volume) of medicines imported to Kyrgyzstan, by therapeutic group and country of origin, for 2013–2015.

To understand exchange rate fluctuation during 2013–2015, monthly data on the exchange rate of the Kyrgyz som to other currencies (US dollar, euro and Russian rouble) were sourced from the Kyrgyz National Bank [62].

### Analyses and validation

For the dataset relating to the medicines reimbursed under the ADP, data were translated into English and cleared for analysis. The data provided on individual medicines were summarised for each INN and grouped into the appropriate therapeutic group (at the first level of the WHO Anatomical Therapeutic and Chemical (ATC) classification system, which describes the main anatomical group, e.g. A – alimentary tract and metabolism). Based on the data on the pharmacy retail prices and reimbursement amounts, co-payments were calculated for each INN at the regional levels. Per prescription co-payments, reimbursement amounts and prices were analysed – in total as well as by ATC group and by region.

The datasets for imported medicines included information on the import batches, such as the medicines, the country of production, the volume contained and the value of the batch. However, approximately 15% of the descriptions of the batches had at least one missing piece of information. Total volumes and values of imported medicines were calculated and analysed in relation to the countries of production.

Given the limitations of the datasets, research was limited to descriptive statistical analyses, and no econometric analysis was performed.

Preliminary analyses were presented and discussed with officials of the MHIF, health financing experts of the WHO Barcelona Office for Health Systems Strengthening, staff of the WHO Country Office in Kyrgyzstan and representatives of international organisations (including the World Bank), with the aims of validating the analysis and receiving comments, which were subsequently incorporated.

## Results

### Co-payments for medicines prescribed and dispensed under the ADP

**Fig. 1:**
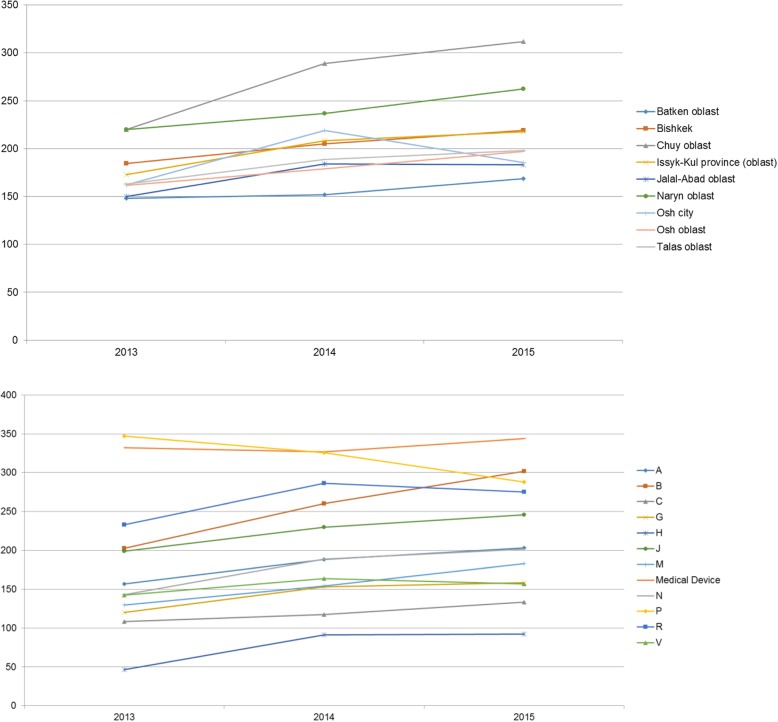
Average co-payments per prescription (expressed in Kyrgyz som) dispensed under the Additional Drug Package, by region (upper panel) and by ATC group (lower panel), 2013–2015

In the study period, co-payments per prescription dispensed under the ADP increased, on average throughout the country, by 22.8%. Across almost all ATC groups, the growth in co-payments was larger in 2014 than in 2015, with decreases in some ATC groups in 2015. For medicines most frequently prescribed (ATC groups B, C and J) the growth rates ranged between 8.3 and 28.4% in 2014 and between 6.9 and 15.9% in 2015. While some regional variation was observed, the majority of Kyrgyz patients faced continuous increases in co-payments for ADP listed medicines. Particularly high growth in co-payments (41.7%) was observed in Chuy oblast (Fig. 1, details in the Additional file 1: Table A1).

**Fig. 2:**
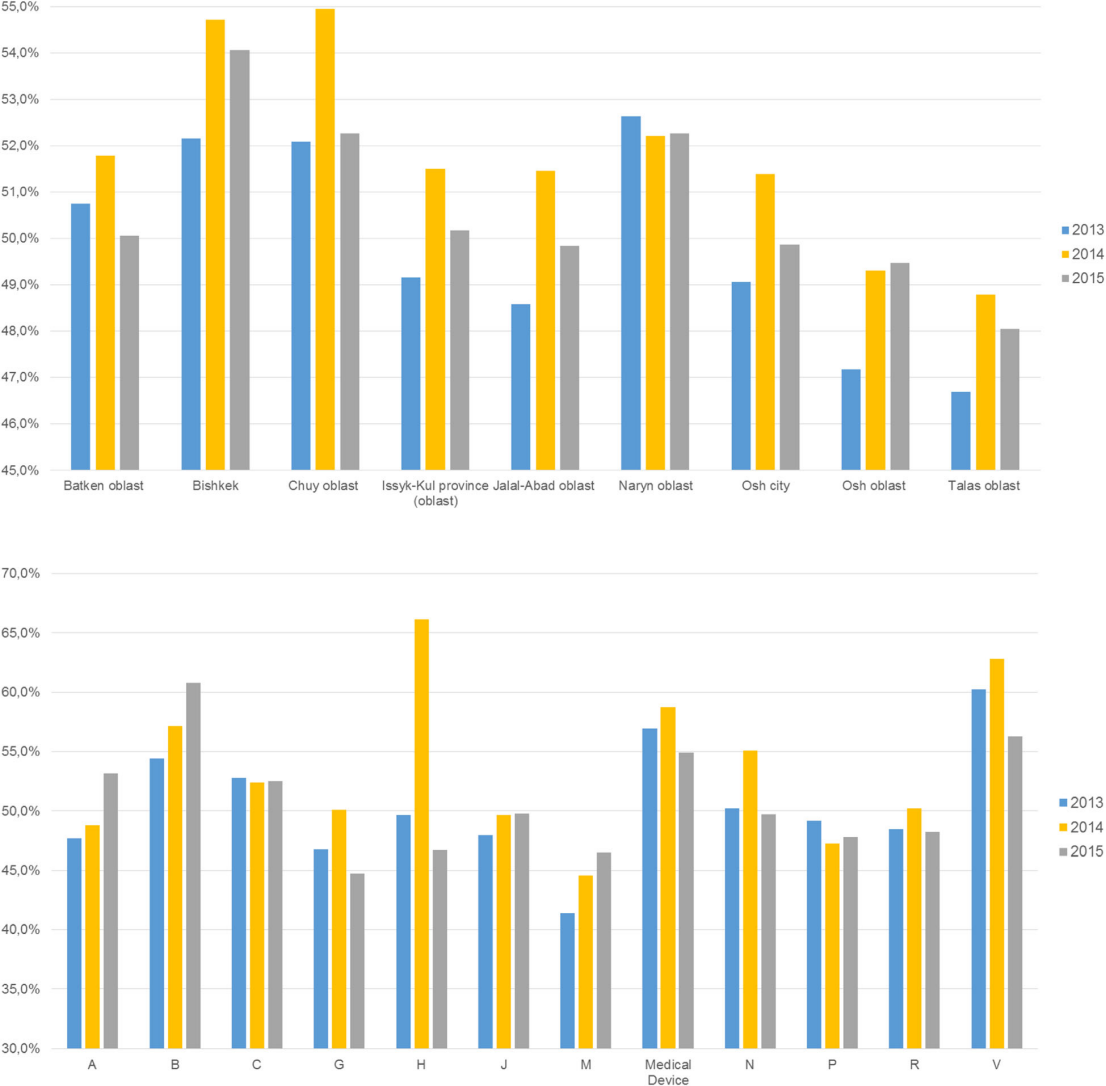
Share of co-payments for medicines prescribed and dispensed (in per cent of pharmacy retail prices) under the Additional Drug Package, by region (upper panel) and by ATC group (lower panel), 2013–2015

On average, patients co-paid at least 49.8% of the pharmacy retail price for ADP medicines in 2013. This amount increased, on average, to 51.8% in 2014 and fell to 50.7% in 2015. Medicines for blood and blood forming organs (ATC code B) had average co-payments of at least 61.8% of the price (Fig. 2, Additional file 1: Table A2)*.*

### Medicines prescribed and dispensed under the ADP in volume and value

While co-payments tended to increase in the observation period, volume data showed a downward trend (on average a 14.3% decrease in the number of medicines prescribed and dispensed under the ADP countrywide). However, the decline was not evenly distributed across the regions and ATC groups, and the extent of decline varied over time. Some regions experienced deeper drops than others; the reductions were particularly considerable in Osh and its surrounding region. Overall, 2014–2015 decreases were lower than 2013–2014 changes, and two regions (Talas oblast, Jalal-Abad oblast) even had more medicines dispensed under the ADP in 2015 compared to 2014. At national level, in ATC groups with higher number of prescriptions, antiinfectives and medicines for the respiratory system experienced large reductions, whereas the number of prescriptions for cardiovascular medicines remained rather stable (Additional file 1: Table A3).

In value, however, the changes showed a different pattern. After a slight decrease in 2014 (− 0.9%), total MHIF expenditures to cover medicines prescribed and dispensed under the ADP increased by 8.5% in 2015. The growth was observed in nearly all ATC groups.

**Fig. 3:**
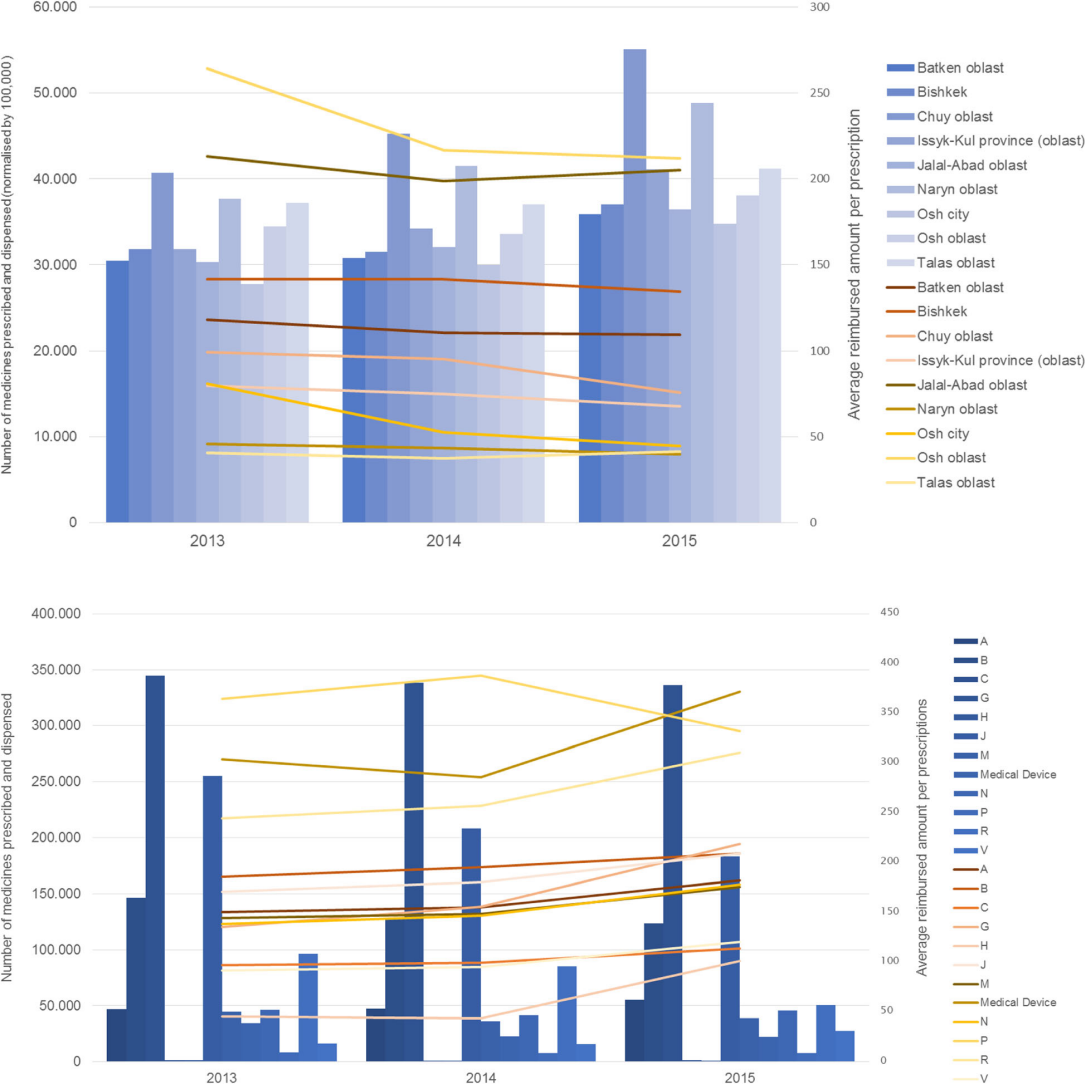
Reimbursed amounts per prescription (expressed in Kyrgyz som) and number of medicines prescribed and dispensed under the Additional Drug Package, by region (upper panel) and by ATC group (lower panel), 2013–2015

Increases in average reimbursed amounts per prescription dispensed under the ADP were even stronger, as a result of the declining number of prescriptions. The amounts reimbursed by MHIF grew by 22% in the study period, with some variation across regions. The increases were largest in Chuy oblast (35.5%), Naryn oblast (29.7%) and Issyk-Kul oblast (29.1%). Talas oblast (10.9%) and Osh oblast (10.5%) experienced lowest increases in comparison. Increases were higher in 2015 compared to 2014 (2013–2014, national average of + 4.8%, 2014–2015, + 16.4%, but decreases in a few regions and ATC groups, Fig. 3, Additional file 1: Table A4).

### Prices of medicines prescribed and dispensed under the ADP

**Fig. 4:**
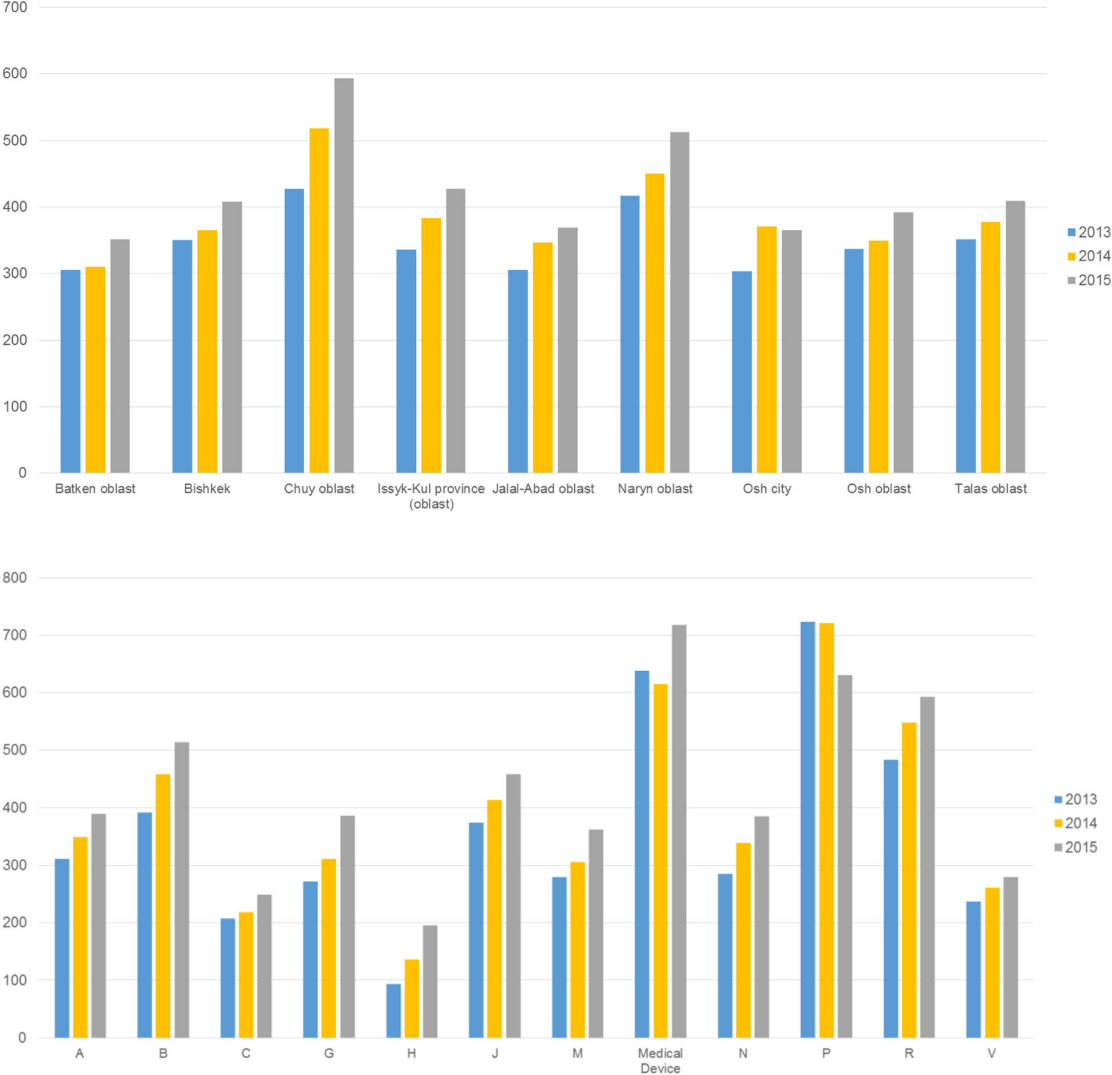
Average prices of medicines prescribed and dispensed under the Additional Drug Package, by region (upper panel) and by ATC group (lower panel), 2013–2015

Average prices per prescription (national average 2015: 425 som) varied between regions, ranging from 352 som in Batken oblast to 593 som in Chuy oblast, and between ATC groups (from 249 som for cardiovascular medicines to 593 som for medicines to treat diseases in the respiratory system; only considering ATC groups whose prescriptions accounted at least 4%). The average prices of medicines prescribed and dispensed under the ADP increased by 22.1% from 2013 and 2015, again with some variation across regions, ATC groups and years. Increases were observed for nearly all ATC groups (exception: antiparasitic products, insecticides and repellents that, however, accounted for few prescriptions) with growth rates ranging from 20.4% for cardiovascular medicines to 35.2% for medicines related to the nervous system (again, only ATC groups with a share of at least 4% of total prescriptions considered; Fig. 4, Additional file 1: Table A5).

### Exchange rate developments

After 2014 the exchange rate of the Kyrgyz som saw a considerable loss in value against the US dollar. Russia is a main trading partner and the crisis in the Russian economy, alongside with a depreciation of the Russian rouble, considerably impacted Kyrgyzstan. While in 2013 the depreciation of Kyrgyz som against the US dollar was still minor, the Kyrgyz currency lost almost half its value against the US dollar in the years 2014 and 2015 (Additional file 1: Table A6).

**Fig. 5:**
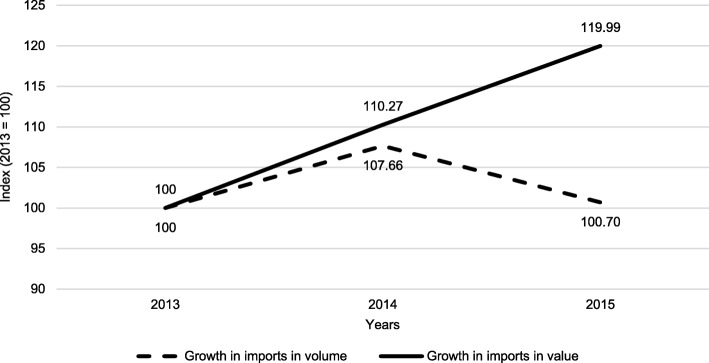
Medicines imported to Kyrgyzstan, in volume and value, 2013–2015

Data analysis of imported medicines showed that, as a result of the exchange rate volatility, in 2015 Kyrgyzstan paid nearly 20% more for approximately the same amount of imported medicines compared to 2013 since in terms of volume the 2015 imports of medicines were at around the same level as of 2013 (Fig. 5).

## Discussion

Since its independence in 1991, Kyrgyzstan has been subject to several reforms that aimed to strengthen the health care system and ensuring a more equitable and affordable access to essential medicines. While progresses have been made over the last two decades [7, 31], there were indications that OOP payments for health care, which had been reduced in earlier times, started to rise again. Household surveys suggested that OOP payments for outpatient medicines, including those subsidised by the benefits package scheme, could be a key driver of private payments [11].

This research confirmed increases in co-payments for medicines funded under the ADP between 2013 and 2015, with variations across regions, therapeutic groups and years.

Kyrgyzstan is among several low- and middle-income countries that have struggled with high OOP payments for medicines (e.g. Pakistan [63], Mongolia [28]). Co-payments for medicines have also been identified as an issue for upper middle- and high-income countries. In Poland, for instance, 14% of households spent more than 10% of their income on medicines in 2000, and the share increased to 18% in 2009 [64]. Evidence on the financial burden of payments for medicines, including its regressive character (i.e. higher share of total expenses for people on lower incomes) in some cases, is available for further high-income countries, such as Austria, Czech Republic, Estonia, Hungary and Latvia [60, 65–69].

According to existing evidence increased co-payments usually led to a fall in public pharmaceutical expenditure and, in most cases, to a reduction in medicine use, possibly combined with reduced medicine adherence [14, 24, 34, 37, 39–41, 44–46, 48, 50, 70]. The data from this research also point to reductions in the number of medicines prescribed and dispensed but public pharmaceutical spending was not reduced in Kyrgyzstan. This rather unusual development might be explained by the increases in prices that appear to have undermined the MHIF’s increased investments. Co-payments risk to increase inequity given its pro-poor and pro-sick effects: increased co-payments were shown to disproportionately shift the financial risk to the very sick and to put people on low incomes at greater risk in terms of poor health outcomes compared to higher-income patients [35].

In comparison to high-income countries with advanced universal health coverage, the financial burden of co-payments for subsidised medicines that patients face is considerably higher in Kyrgyzstan, as a recent WHO report showed [60]. The study compared the burden of co-payments for patients in nine countries in the WHO European Region, including Albania, Kyrgyzstan, and wealthy economies such as Austria, France, Germany and Sweden. Co-payments as a share of the minimum wage were highest in Kyrgyzstan for the medicines studied: in 2017, Kyrgyz patients paid 9% of the minimum wage for a one-month pack of generic amlodipine (a cardiovascular medicine), and 2–4% for generic and originator salbutamol needed for 1 month of asthma treatment. The findings of that study pointed to inequity, since Kyrgyz legislation did not define any exemptions or reductions that could ease the burden for population groups subject to vulnerability. Similar conclusions on inequity were drawn in a recent Organisation for Economic Co-operation and Development (OECD) review: it concluded that Kyrgyz co-payment regulation in health care (not necessarily focused on medicines) was not designed to promote equity, and targeted poor and rural populations ineffectively [8]. This adds to the policy recommendation expressed in several reports that co-payments should be designed in a way that exempts specific population groups [34, 44, 60, 68, 71, 72].

Findings on the extent and evolutions of co-payments, reimbursement amounts and prescriptions under the ADP showed regional variation in Kyrgyzstan. Chuy oblast (surrounding the capital city Bishkek) had the highest increases in co-payments (+ 41.7% in the study period; + 31.3% 2013–14, + 7.9% 2014–15) that nearly doubled the average countrywide growth. At the same time, Chuy oblast was the region with highest average reimbursement amounts per prescription (2015, 276 som in Chuy oblast, 205 som on national average) as well as the highest increases in reimbursement amounts (2013–15, + 35,5% in Chuy oblast, + 22% on national average). This suggests possible inefficiencies as public funding is provided by the MHIF but is apparently not effective in reducing the financial burden for the patients. In 2015, 20% fewer prescriptions were dispensed in Chuy oblast compared to the previous year; this could be an indication for patients not filling prescriptions due to financial restrictions, as known from other studies on the effects of OOP spending, including co-payments, on medicines [34–36, 40, 41, 43, 44, 48, 50]. In fact, the decrease in medicines prescribed and dispensed in Chuy oblast was the largest of all regions in 2015, and this region also showed the highest average price per prescription in the years studied and the highest growth rates in prices during this period.

Another case of interest is Osh city, the second largest town of Kyrgyzstan. Co-payments per prescription increased by 34.8% in 2014 (highest growth rate of all regions; national average: + 17.8%) but decreased by 15.3% in 2015 (the only region with substantial decreases, most other regions had increases; national average: + 4.3%). Though, at first glance, this could be interpreted as indication for more affordable access to medicines, caution has to be exercised when drawing this conclusion. It is to note that Osh city was the region with highest decreases in prescriptions dispensed in the study period (2013–2014, ^-^ − 35.3%, highest decrease of all regions, national average, − 10.5%, 2014–15, − 15%, second largest decrease after Chuy oblast, national average, − 4.2%). Information is lacking to interpret the data but a decrease in health care utilisation, as suggested by a reduced number of prescriptions, might be an indication of unaffordability, at least for some population groups, which may occur despite decreasing co-payments. In Australia, a study [73] on OOP spending on cancer showed that Indigenous people with cancer accessed fewer subsidized services even if they were charged lower co-payments, so specific protection mechanisms would be required for some population groups to increase health service utilisation.

The MHIF grants higher ‘baseline prices’ (reimbursement amounts) for rural areas, with the aim of adjusting for regional variation and thus facilitating lower co-payments. Data analysis showed that this policy was partially effective: While Issyk-Kul oblast (10 inhabitants / km^2^) and Talas oblast (18 inhabitants / km^2^) had lower shares of co-payments, Naryn oblast (6 inhabitants / km^2^, thus the least populated region and also considered the poorest region in the country) had consistently higher co-payments (average co-payment share of at least 52%, national averages around 50% in 2013–2015).

The findings suggest regional inequities. More analyses are needed to understand in detail the developments in the regions. However, given the lack of quantitative data, these investigations would probably need to be based on qualitative research such as semi-structured interviews in the regions [74]. At a macro level, the study provides evidence about growth in co-payments for medicines throughout the country between 2013 and 2015 even though MHIF expenditure to fund medicines was extended in 2015. While fewer prescriptions were dispensed in pharmacies in the study period, average reimbursed amounts – and average co-payments – per prescription increased in nearly all ATC groups. Thus, more money (both by the public payer and the patients) was spent while volume decreased (fewer prescriptions). These developments suggest increases in the prices of ADP-listed medicines.

Some information on medicine prices and their development can be obtained from a survey of the Medicines Transparency Alliance (MeTA) project [57] that was conducted using the WHO / Health Action International (HAI) methodology to measure medicine prices, availability, affordability and price components in Kyrgyzstan in 2015 and to compare them to 2005 and 2010 data gained using the same methodology [75]. Median prices for the majority of generics in the private sector were shown to have declined during the last decade: while the change in median prices was minor if 2015 data were compared to 2005 (median price ratio / MPR 2005 = 1.29; MPR 2015: 1.04), median prices of these medicines decreased considerably compared to prices in 2010 (MPR 2010 = 2.36)^,^. However, prices of both originator and generic medicines remained high in international comparison: the median of MPR for generic medicines was 2.17 times the international reference prices. Though average patient payments for a treatment course of priority health conditions by the lowest paid population declined from seven daily wages in 2005 to two in 2015, treatment of acute and chronic conditions remained expensive and could require up to 15 daily wages [57].

Some of the rather positive trends that the MeTA price survey showed have to be interpreted with caution, since the improvements relate to longer time periods (5 and 10 years, respectively), and developments in between (e.g. annual changes) are not known. While differences in methodology (different baskets of medicines surveyed, different time lines) of the MeTA survey and this research limit comparability, both studies concluded that parts of outpatient medicines were unaffordable for the Kyrgyz population in 2015. Unaffordability of essential medicines is a barrier to access to medicines in many countries worldwide [76–80].

Throughout the regions and the therapeutic groups, increases in co-payments and prices in 2013–2014 were considerably higher than in 2014–2015. 2014 was the year when the Kyrgyz som started to devaluate importantly against the US dollar, and, at the same time, from mid-2014, the Russian rouble devaluated against the Kyrgyz som. This points to an impact of the depreciation of the national currency, in response to the economic turndown, as an additional driver for the increases in prices and in co-payments that are linked to the prices. Kyrgyzstan’s high dependency on medicine imports has aggravated the situation. The impact of economic crisis and a depreciation of a country’s national currency on medicine prices, particularly in unregulated settings has been observed in other countries as well (e.g. Pakistan [81], Argentina [82]).

In addition, the absence of medicine price regulation probably worsened the effects of the currency depreciation. Evidence from several countries shows that patients have been confronted with unaffordable, high prices in settings without price regulation [75–78, 83–89]. Frequently, add-ons on prices in the supply chain also account considerably for final unaffordable prices. In Kyrgyzstan, wholesale and pharmacy mark-ups are not regulated. A 2007 survey of a pharmacy network in remote Kyrgyzstan revealed retail mark-ups in the range of 32 to 244% for the network’s top 50 medicines (i.e. those that accounted for more than 50% of their profits) [9].

### Policy implications

The findings of this research on Kyrgyzstan identified high medicine prices as a major explanatory factor for high and increasing OOP payments for medicines. There is evidence that price control can contribute to lower and more affordable medicine prices [70, 83, 90–92]. This benefits patients who have to pay fully out of pocket as well as those who have access to subsidised medicines included in the benefit package schemes. Thus, the study confirms a need for price regulation for medicines. Price control should address all levels in the supply chain and include regulation of distribution mark-ups [83], as it has been stressed that ‘regulation of mark-ups without regulation of either the manufacturer’s selling price or the retail selling price is unlikely to lead to reduced medicine prices’ [93]. Kyrgyzstan’s topography as a country with large rural, mountainous areas could be taken into consideration with a geographical differentiation that would allow higher distribution mark-ups for remote areas, in order to provide an incentive to supply these regions and thus ensure more equitable accessibility throughout the country.

The introduction of medicine price regulation should be accompanied by improvements in transparency and data availability. For the time being, the MHIF calculates the ‘baseline price’ that it reimburses based on price information requested from some wholesalers. Thus, the public payer depends on the goodwill of private sector representatives to share data, and there is no opportunity to validate the data ex ante. In this respect, undertaking regular medicine price surveys – such as those using the WHO/HAI methodology [75] – could help to give a more comprehensive picture of the market. Furthermore, the eHealth strategy 2015–2020 that Kyrgyzstan adopted in 2015 commits to the establishment of a comprehensive medicine information system that should cover ‘all aspects of medicine provision from product registration to sale and use’ [94]. This could be helpful in monitoring and evaluating the impact of medicine price regulation and any further pharmaceutical policies to be implemented.

In addition, further policies could also contribute to improve equity in medicine use and reduce OOP payments for medicines. These include ensuring sufficient public funding of the health care system; inclusion of medicines that serve patients’ priority health needs, with careful selection based on their therapeutic benefits and cost-effectiveness; enhancing the uptake of generics and lower-priced medicines; implementing a strategic design of the pharmaceutical reimbursement policy framework and formulating a co-payment regulation containing reductions and exemptions for populations that require stronger financial protection since patient payments are likely to lead to adverse health outcomes among people on low incomes, older people and patients with chronic conditions, partly through reduced adherence to essential medicines [60, 71, 72, 95–99]. A combination of policies is favourable [83].

In this respect, it is notable that Kyrgyzstan’s ADP includes a rather limited number of outpatient medicines (58 INNs in 2015). Capped prescription budgets of family group medical practices also contribute to rationing at the prescriber level. These restrictions resulted in a total of 1.2 million prescriptions being processed in Kyrgyzstan in 2017, while an estimated 1 million people would require regular prescriptions for hypertension alone [12].

### Limitations and research implications

The study has some limitations. The MHIF dataset used for the analysis only contained medicines prescribed and dispensed: no evidence was collected on possible prescriptions that had not been filled. Such information could have provided indications of possible non-affordability of medicines. Furthermore, the available data only allowed analysis of formal co-payments for medicines listed in the ADP. Any co-payments under the SGBP (which also includes a few medicines for outpatient use) could not be assessed, and informal payments for medicines were not addressed in this study. No econometric analyses were performed.

In the light of these limitations, there is room for further research on outpatient medicines included in the SGBP and on informal co-payments as well as on prescriptions that were not filled. Analyses on regional inequities could provide further knowledge. Additional methods, including qualitative ones (e.g. interviews) and on-site data collection, would be required to address these research questions since human resources of the data supplying institutions are limited.

Despite the methodological limitations, however, the study provides new valuable information about patient co-payments and prices for medicines in Kyrgyzstan. The analysis confirmed anecdotal evidence as well as trends shown in previous research [11]. The study is the first investigation of co-payments of outpatient reimbursable medicines in Kyrgyzstan. While previous research used to be based on household surveys, this study analysed primary data received from the public payer and the regulatory authority.

## Conclusions

Co-payments for outpatient ADP-listed medicines increased between 2013 and 2015. Growth in co-payments was inequitable, with variations across regions, therapeutic groups and years. Data analysis suggests that the growth in medicine prices was a major driver for rising co-payments in Kyrgyzstan. The price increases were partly attributable to a major devaluation of the Kyrgyz currency – as such, they were unavoidable. A further explanation is the absence of medicine price regulation, which aggravated the effects of the devaluation. The substantial sums that Kyrgyz patients have been spending on outpatient reimbursable medicines constitute a significant barrier to access, and they risk undermining the progresses that Kyrgyzstan has made in strengthening of the health care system and towards achieving universal health coverage. Thus, it is recommended that the Kyrgyz government should move forward in implementing medicine price regulation. In the development of the pharmaceutical pricing policy framework, control of prices in the supply chain and incentives for lower-priced medicines should also be considered and mechanisms to access, survey and analyse medicine price information as well as to monitor and publish the progresses made through the price regulation should be installed. With regard to co-payment regulation, exemptions for specific population groups – in particular people on low incomes – would be beneficial in ensuring more affordable and equitable access to essential medicines.

## Additional file

Additional file 1: **Table A1.** Average co-payments per prescription dispensed under the ADP, by region and by ATC group, 2013–2015. **Table A2.** Share of co-payments for medicines prescribed and dispensed under the ADP, by region and by ATC group, 2013–2015. **Table A3.** Number of medicines prescribed and dispensed under the ADP, by region and by ATC group, 2013–2015. **Table A4.** Average amounts reimbursed per prescription dispensed under the APD, by region and by ATC group, 2013–2015. **Table A5.** Average prices of medicines prescribed and dispensed under the ADP, by region and by ATC group, 2013–2015. **Table A6.** Exchange rates developments of the Kyrgyz som in comparison to the US dollar, the euro and Russian rouble. (DOCX 83 kb)

## Data Availability

Data generated and analysed during this study are included in this published article and its Supplementary information file.

## Abbreviations

ADP: Additional Drug Package
ATC: Anatomical Therapeutic Chemical
HAI: Health Action International
INN: International non-proprietary name
MeTA: Medicines Transparency Alliance
MHIF: Mandatory Health Insurance Fund
MPR: Median price ratio
OECD: Organisation for Economic Co-operation and Development
OOP: Out-of-pocket
SGBP: State Guaranteed Benefit Programme
US$: United States dollar
WHO: World Health Organization
