# Supplementary material for: Affordable and equitable access to subsidised outpatient medicines? Analysis of co-payments under the Additional Drug Package in Kyrgyzstan
Source: Int J Equity Health. 2019 Jun 13;18:89. doi: 10.1186/s12939-019-0990-6 (PMC6567501; doi:10.1186/s12939-019-0990-6)
Supplement: Supplementary file 1 — Table A1. Average co-payments per prescription dispensed under the ADP, by region and by ATC group, 2013–2015. Table A2. Share of co-payments for medicines prescribed and dispensed under the ADP, by region and by ATC group, 2013–2015. Table A3. Number of medicines prescribed and dispensed under the ADP, by region and by ATC group, 2013–2015. Table A4. Average amounts reimbursed per prescription dispensed under the APD, by region and by ATC group, 2013–2015. Table A5. Average prices of medicines prescribed and dispensed under the ADP, by region and by ATC group, 2013–2015. Table A6. Exchange rates developments of the Kyrgyz som in comparison to the US dollar, the euro and Russian rouble. (DOCX 83 kb) [file 12939_2019_990_MOESM1_ESM.docx]

**Additional file 1**

**Table A1**

Average co-payments per prescription dispensed under the ADP, by region and by ATC group, 2013–2015

| **Category** | **% of prescr.** | **Co-payment per prescription (KGS)** | | | **Changes in average co-payments (in %)** | | |
| --- | --- | --- | --- | --- | --- | --- | --- |
|  | **(2015)** | **2013** | **2014** | **2015** | **2013–15** | **2013–14** | **2014–15** |
| **Region** | | | | | | | |
| Batken oblast | 11.8 | 148.04 | 152.06 | 168.83 | 14.04 | 2.72 | 11.03 |
| Bishkek | 14.4 | 184.67 | 204.97 | 219.26 | 18.73 | 10.99 | 6.97 |
| Chuy oblast | 8.2 | 220.11 | 289.00 | 311.83 | 41.67 | 31.30 | 7.90 |
| Issyk-Kul oblast | 7.3 | 172.66 | 208.20 | 217.49 | 25.96 | 20.58 | 4.46 |
| Jalal-Abad oblast | 22.1 | 149.83 | 184.10 | 183.25 | 22.30 | 22.87 | -0.46 |
| Naryn oblast | 4.3 | 220.09 | 236.76 | 262.30 | 19.18 | 7.57 | 10.79 |
| Osh city | 4.8 | 162.36 | 218.87 | 185.33 | 14.15 | 34.80 | -15.32 |
| Osh oblast | 22.8 | 161.83 | 178.78 | 196.92 | 21.68 | 10.47 | 10.15 |
| Talas oblast | 4.4 | 163.25 | 188.80 | 198.17 | 21.39 | 15.65 | 4.96 |
| **ATC groups** | | | | | | | |
| A – Alimentary tract and metabolism | 6.20 | 156.76 | 188.44 | 203.14 | 29.59 | 20.21 | 7.80 |
| B – Blood and blood forming organs | 13.82 | 202.69 | 260.34 | 301.78 | 48.88 | 28.44 | 15.92 |
| C – Cardiovascular system | 37.64 | 108.44 | 117.38 | 133.48 | 23.09 | 8.25 | 13.71 |
| G – Genito-urinary system and sex hormones | 0.14 | 119.89 | 153.12 | 158.60 | 32.29 | 27.72 | 3.58 |
| H – Systemic hormonal preparations. excluding sex hormones and insulins | 0.10 | 46.43 | 91.13 | 92.52 | 99.29 | 96.28 | 1.53 |
| J – Antiinfectives for systemic use | 20.52 | 198.86 | 229.87 | 245.66 | 23.53 | 15.59 | 6.87 |
| M – Musculo-skeletal system | 4.35 | 129.82 | 154.39 | 183.01 | 40.97 | 18.93 | 18.53 |
| N – Nervous system | 5.11 | 143.04 | 188.76 | 201.69 | 41.00 | 31.96 | 6.85 |
| P – Antiparasitic products, insecticides and repellents | 0.88 | 347.11 | 325.63 | 287.87 | -17.07 | -6.19 | -11.60 |
| R – Respiratory system | 5.68 | 233.03 | 286.31 | 274.92 | 17.98 | 22.86 | -3.98 |
| V - Various | 3.07 | 142.35 | 163.61 | 156.90 | 10.21 | 14.93 | -4.11 |
| Medical devices | 2.49 | 332.05 | 326.83 | 344.01 | 3.60 | -1.57 | 5.26 |
| **Total** | **-** | **175.76** | **206.95** | **215.78** | **22.77** | **17.75** | **4.27** |

ADP: Additional Drug Package; ATC: Anatomical Therapeutic and Chemical classification; KGS: Kyrgyz som; prescr.: prescriptions

**Table A2**

Share of co-payments for medicines prescribed and dispensed under the ADP, by region and by ATC group, 2013–2015

| **Category** | **% of prescr.** | **Average share of co-payment (in %)** | | | **Changes in average share of co-payment (in %)** | | |
| --- | --- | --- | --- | --- | --- | --- | --- |
|  | **2015** | **2013** | **2014** | **2015** | **2013–15** | **2013–14** | **2014–15** |
| **Region** | | | | | | | |
| Batken oblast | 14.4 | 50.75 | 51.78 | 50.06 | -1.34 | 2.04 | -3.32 |
| Bishkek | 8.2 | 52.16 | 54.72 | 54.06 | 3.64 | 4.91 | -1.21 |
| Chuy oblast | 7.3 | 52.09 | 54.95 | 52.27 | 0.35 | 5.49 | -4.88 |
| Issyk-Kul oblast | 22.1 | 49.16 | 51.51 | 50.18 | 2.07 | 4.78 | -2.58 |
| Jalal-Abad oblast | 4.3 | 48.58 | 51.45 | 49.84 | 2.58 | 5.91 | -3.14 |
| Naryn oblast | 4.8 | 52.64 | 52.21 | 52.27 | -0.70 | -0.81 | 0.11 |
| Osh city | 22.8 | 49.06 | 51.39 | 49.87 | 1.65 | 4.74 | -2.95 |
| Osh oblast | 4.4 | 47.17 | 49.31 | 49.47 | 4.88 | 4.52 | 0.34 |
| Talas oblast | 11.8 | 46.70 | 48.78 | 48.05 | 2.90 | 4.47 | -1.50 |
| **ATC group** | | | | | | | |
| A – Alimentary tract and metabolism | 6.20 | 48.71 | 49.95 | 54.89 | 12.47 | 2.87 | 9.33 |
| B – Blood and blood forming organs | 13.82 | 55.30 | 57.52 | 61.78 | 11.12 | 3.77 | 7.08 |
| C – Cardiovascular system | 37.64 | 53.54 | 53.20 | 53.67 | 0.96 | 0.53 | 0.43 |
| G – Genito-urinary system and sex hormones | 0.14 | 48.51 | 50.76 | 45.92 | -5.70 | 5.08 | -10.26 |
| H – Systemic hormonal preparations, excluding sex hormones and insulins | 0.10 | 50.56 | 66.66 | 47.09 | -3.26 | 35.87 | -28.80 |
| J – Antiinfectives for systemic use | 20.52 | 49.07 | 50.11 | 50.52 | 3.44 | 3.52 | -0.07 |
| M – Musculo-skeletal system | 4.35 | 42.09 | 45.02 | 46.96 | 14.20 | 11.14 | 2.75 |
| N – Nervous system | 5.11 | 50.87 | 55.80 | 50.55 | -1.48 | 9.13 | -9.72 |
| P – Antiparasitic products, insecticides and repellents | 0.88 | 50.34 | 48.09 | 48.31 | -1.95 | -3.89 | 2.02 |
| R – Respiratory system | 5.68 | 49.20 | 50.75 | 49.16 | -0.88 | 3.03 | -3.79 |
| V - Various | 3.07 | 60.87 | 63.31 | 56.67 | -7.21 | 3.52 | -10.36 |
| Medical devices | 2.49 | 57.26 | 59.07 | 55.24 | -2.39 | 3.44 | -5.64 |
| **Total** | **-** | **49.81** | **51.77** | **50.67** | **1.93** | **4.03** | **-2.02** |

ADP: Additional Drug Package; ATC: Anatomical Therapeutic and Chemical classification, prescr: prescriptions

**Table A3**

Number of medicines prescribed and dispensed under the ADP, by region and by ATC group, 2013–2015

| **Category** | **Number in medicines prescribed and dispensed** | | | **Changes in number of in medicines prescribed and dispensed (in %)** | | |
| --- | --- | --- | --- | --- | --- | --- |
|  | **2013** | **2014** | **2015** | **2013–15** | **2013–14** | **2014–15** |
| **Regions** | | | | | | |
| Batken oblast | 113551 | 106013 | 105016 | -7.52 | -6.64 | -0.94 |
| Bishkek | 136241 | 136240 | 129083 | -5.25 | 0.00 | -5.25 |
| Chuy oblast | 95535 | 91399 | 72818 | -23.78 | -4.33 | -20.33 |
| Issyk-Kul oblast | 76766 | 72007 | 64920 | -15.43 | -6.20 | -9.84 |
| Jalal-Abad oblast | 204915 | 190901 | 197078 | -3.82 | -6.84 | 3.24 |
| Naryn oblast | 43994 | 41757 | 38280 | -12.99 | -5.08 | -8.33 |
| Osh city | 77696 | 50298 | 42744 | -44.99 | -35.26 | -15.02 |
| Osh oblast | 254088 | 208170 | 203724 | -19.82 | -18.07 | -2.14 |
| Talas oblast | 38991 | 35999 | 39700 | 1.82 | -7.67 | 10.28 |
| **ATC group** | | | | | | |
| A – Alimentary tract and metabolism | 46728 | 47386 | 55383 | 18.52 | 1.41 | 16.88 |
| B – Blood and blood forming organs | 146496 | 127412 | 123460 | -15.72 | -13.03 | -3.10 |
| C – Cardiovascular system | 344436 | 338055 | 336269 | -2.37 | -1.85 | -0.53 |
| G – Genito-urinary system and sex hormones | 1290 | 1006 | 1276 | -1.09 | -22.02 | 26.84 |
| H – Systemic hormonal preparations, excluding sex hormones and insulins | 1358 | 940 | 866 | -36.23 | -30.78 | -7.87 |
| J – Antiinfectives for systemic use | 254995 | 208143 | 183283 | -28.12 | -18.37 | -11.94 |
| M – Musculo-skeletal system | 44746 | 36253 | 38854 | -13.17 | -18.98 | 7.17 |
| N – Nervous system | 46071 | 41612 | 45661 | -0.89 | -9.68 | 9.73 |
| P – Antiparasitic products, insecticides and repellents | 8502 | 7734 | 7891 | -7.19 | -9.03 | 2.03 |
| R – Respiratory system | 96287 | 85368 | 50700 | -47.34 | -11.34 | -40.61 |
| V - Various | 16419 | 15893 | 27440 | 67.12 | -3.20 | 72.65 |
| Medical devices | 34449 | 22982 | 22280 | -35.32 | -33.29 | -3.05 |
| **Total** | **1041777** | **932784** | **893363** | **-14.25** | **-10.46** | **-4.23** |

ADP: Additional Drug Package; ATC: Anatomical Therapeutic and Chemical classification

**Table A4**

Average amounts reimbursed per prescription dispensed under the APD, by region and by ATC group, 2013–2015

| **Category** | **Average amounts reimbursed / prescription (KGS)** | | | | | | **Changes in average amounts reimbursed (in %)** | | |
| --- | --- | --- | --- | --- | --- | --- | --- | --- | --- |
|  | **2013** | | **2014** | | **2015** | | **2013–15** | **2013–14** | **2014–15** |
| **Regions** | | | | | | | | | |
| Batken oblast | 152.24 | | 154.06 | | 179.60 | | 17.97 | 1.19 | 16.58 |
| Bishkek | 159.17 | | 157.65 | | 185.29 | | 16.41 | -0.95 | 17.53 |
| Chuy oblast | 203.38 | | 226.20 | | 275.59 | | 35.50 | 11.22 | 21.83 |
| Issyk-Kul oblast | 159.36 | | 171.24 | | 205.67 | | 29.07 | 7.46 | 20.11 |
| Jalal-Abad oblast | 151.53 | | 160.45 | | 182.47 | | 20.41 | 5.89 | 13.72 |
| Naryn oblast | 188.31 | | 207.60 | | 244.28 | | 29.72 | 10.25 | 17.67 |
| Osh city | 138.94 | | 150.14 | | 173.76 | | 25.06 | 8.06 | 15.73 |
| Osh oblast | 172.41 | | 167.84 | | 190.44 | | 10.46 | -2.65 | 13.46 |
| Talas oblast | 185.76 | | 185.14 | | 205.99 | | 10.89 | -0.34 | 11.26 |
| **ATC groups** | | | | | | | | | |
| A – Alimentary tract and metabolism | 150.46 | 155.14 | | 181.94 | | 20.92 | | 3.11 | 17.28 |
| B – Blood and blood forming organs | 185.95 | 195.18 | | 209.35 | | 12.58 | | 4.96 | 7.26 |
| C – Cardiovascular system | 96.78 | 99.52 | | 113.84 | | 17.63 | | 2.83 | 14.40 |
| G – Genito-urinary system and sex hormones | 135.65 | 155.69 | | 218.83 | | 61.32 | | 14.78 | 40.55 |
| H – Systemic hormonal preparations, excluding sex hormones and insulins | 45.49 | 43.82 | | 101.12 | | 122.30 | | -3.68 | 130.79 |
| J – Antiinfectives for systemic use | 170.53 | 180.42 | | 209.27 | | 22.72 | | 5.80 | 16.00 |
| M – Musculo-skeletal system | 144.20 | 148.58 | | 175.32 | | 21.58 | | 3.04 | 18.00 |
| N – Nervous system | 138.24 | 146.47 | | 178.10 | | 28.83 | | 5.96 | 21.59 |
| P – Antiparasitic products, insecticides and repellents | 364.59 | 387.89 | | 332.10 | | -8.91 | | 6.39 | -14.38 |
| R – Respiratory system | 244.44 | 257.07 | | 310.65 | | 27.09 | | 5.17 | 20.84 |
| V - Various | 91.85 | 95.07 | | 120.57 | | 31.27 | | 3.51 | 26.82 |
| Medical devices | 303.99 | 286.01 | | 371.65 | | 22.26 | | -5.91 | 29.94 |
| **Total** | **167.77** | **175.82** | | **204.66** | | **21.98** | | **4.80** | **16.40** |

ADP: Additional Drug Package; ATC: Anatomical Therapeutic and Chemical classification; KGS: Kyrgyz som

**Table A5**

Average prices of medicines prescribed and dispensed under the ADP, by region and by ATC group, 2013–2015

| **Category** | **% of prescr.** | **Average price per prescription (KGS)** | | | **Changes (in %)** | | |
| --- | --- | --- | --- | --- | --- | --- | --- |
|  | **2015** | **2013** | **2014** | **2015** | **2013–15** | **2013–14** | **2014–15** |
| **Region** | | | | | | | |
| Batken oblast | 14.4 | 305.44 | 310.15 | 351.97 | 15.23 | 1.54 | 13.48 |
| Bishkek | 8.2 | 350.38 | 365.34 | 408.09 | 16.47 | 4.27 | 11.70 |
| Chuy oblast | 7.3 | 427.10 | 518.37 | 593.49 | 38.96 | 21.37 | 14.49 |
| Issyk-Kul oblast | 22.1 | 335.98 | 383.08 | 427.04 | 27.10 | 14.02 | 11.48 |
| Jalal-Abad oblast | 4.3 | 305.90 | 346.94 | 368.52 | 20.47 | 13.42 | 6.22 |
| Naryn oblast | 4.8 | 416.52 | 450.04 | 512.96 | 23.15 | 8.05 | 13.98 |
| Osh city | 22.8 | 303.97 | 371.19 | 364.64 | 19.96 | 22.12 | -1.77 |
| Osh oblast | 4.4 | 337.37 | 349.48 | 391.53 | 16.05 | 3.59 | 12.03 |
| Talas oblast | 11.8 | 351.72 | 377.67 | 408.70 | 16.20 | 7.38 | 8.22 |
| **ATC group** | | | | | | | |
| A – Alimentary tract and metabolism | 6.20 | 311.03 | 348.38 | 389.11 | 25.10 | 12.01 | 11.69 |
| B – Blood and blood forming organs | 13.82 | 391.23 | 457.87 | 514.27 | 31.45 | 17.03 | 12.32 |
| C – Cardiovascular system | 37.64 | 207.07 | 218.64 | 249.26 | 20.37 | 5.59 | 14.01 |
| G – Genito-urinary system and sex hormones | 0.14 | 272.08 | 311.34 | 385.86 | 41.82 | 14.43 | 23.94 |
| H – Systemic hormonal preparations, excluding sex hormones and insulins | 0.10 | 93.67 | 136.01 | 195.32 | 108.51 | 45.20 | 43.61 |
| J – Antiinfectives for systemic use | 20.52 | 373.72 | 413.20 | 458.52 | 22.69 | 10.56 | 10.97 |
| M – Musculo-skeletal system | 4.35 | 279.44 | 306.16 | 362.12 | 29.59 | 9.56 | 18.28 |
| N – Nervous system | 5.11 | 284.73 | 339.26 | 384.82 | 35.15 | 19.15 | 13.43 |
| P – Antiparasitic products, insecticides and repellents | 0.88 | 723.07 | 721.27 | 629.76 | -12.91 | -0.25 | -12.69 |
| R – Respiratory system | 5.68 | 483.73 | 547.93 | 593.22 | 22.63 | 13.27 | 8.27 |
| V - Various | 3.07 | 236.67 | 260.85 | 279.42 | 18.06 | 10.21 | 7.12 |
| Medical devices | 2.49 | 638.44 | 614.45 | 718.12 | 12.48 | -3.76 | 16.87 |
| **Total** | **-** | **348.03** | **386.16** | **424.92** | **22.09** | **10.96** | **10.04** |

ADP: Additional Drug Package; ATC: Anatomical Therapeutic and Chemical classification, KGS = Kyrgyz som, prescr.: prescriptions

**Table A6**

Exchange rates developments of the Kyrgyz som in comparison to the US dollar, the euro and Russian rouble

| **Date** | **US dollar** | **euro** | **Russian rouble** | **Kyrgyz som** |
| --- | --- | --- | --- | --- |
| **01.01.2013** | 47.3868 | 62.5293 | 1.5602 | 0.3144 |
| **01.02.2013** | 47.7948 | 64.8313 | 1.5923 | 0.3169 |
| **01.03.2013** | 47.5400 | 62.4699 | 1.5581 | 0.3162 |
| **01.04.2013** | 47.9610 | 61.4884 | 1.5430 | 0.3180 |
| **01.05.2013** | 48.1235 | 62.8758 | 1.5502 | 0.3182 |
| **01.06.2013** | 48.2702 | 62.8213 | 1.5180 | 0.3189 |
| **01.07.2013** | 48.6277 | 63.4543 | 1.4867 | 0.3207 |
| **01.08.2013** | 48.9454 | 65.0411 | 1.4817 | 0.3189 |
| **01.09.2013** | 48.7243 | 64.5305 | 1.4655 | 0.3189 |
| **01.10.2013** | 48.6561 | 65.6784 | 1.4979 | 0.3163 |
| **01.11.2013** | 48.5015 | 66.4980 | 1.5121 | 0.3145 |
| **01.12.2013** | 48.9590 | 66.6234 | 1.4750 | 0.3186 |
| **01.01.2014** | 49.1894 | 67.7313 | 1.5029 | 0.3193 |
| **01.02.2014** | 50.3893 | 68.3002 | 1.4323 | 0.3240 |
| **01.03.2014** | 53.9590 | 73.9994 | 1.4912 | 0.2931 |
| **01.04.2014** | 54.4999 | 74.8692 | 1.3711 | 0.2994 |
| **01.05.2014** | 53.8692 | 74.3099 | 1.5080 | 0.2959 |
| **01.06.2014** | 52.0806 | 70.8843 | 1.4994 | 0.2838 |
| **01.07.2014** | 52.0882 | 71.1316 | 1.5391 | 0.2838 |
| **01.08.2014** | 51.6978 | 69.2337 | 1.4586 | 0.2821 |
| **01.09.2014** | 52.9561 | 69.8517 | 1.4339 | 0.2910 |
| **01.10.2014** | 54.3991 | 68.8937 | 1.3813 | 0.2991 |
| **01.11.2014** | 57.5712 | 72.3900 | 1.3720 | 0.3183 |
| **01.12.2014** | 57.5957 | 71.6433 | 1.1677 | 0.3184 |
| **01.01.2015** | 58.8956 | 71.5847 | 1.0469 | 0.3230 |
| **01.02.2015** | 59.8100 | 67.8395 | 0.8677 | 0.3243 |
| **01.03.2015** | 61.2914 | 68.7414 | 1.0003 | 0.3312 |
| **01.04.2015** | 63.8889 | 68.4889 | 1.1082 | 0.3441 |
| **01.05.2015** | 59.6412 | 66.8548 | 1.1663 | 0.3210 |
| **01.06.2015** | 58.1472 | 63.7119 | 1.0977 | 0.3127 |
| **01.07.2015** | 62.1422 | 69.2388 | 1.1128 | 0.3337 |
| **01.08.2015** | 61.1094 | 66.8354 | 1.0127 | 0.3260 |
| **01.09.2015** | 65.0953 | 73.4991 | 0.9792 | 0.2739 |
| **01.10.2015** | 68.8248 | 77.2730 | 1.0470 | 0.2541 |
| **01.11.2015** | 69.6980 | 76.7131 | 1.0827 | 0.2497 |
| **01.12.2015** | 75.8648 | 80.2384 | 1.1368 | 0.2468 |

Data sourced from the National Bank of the Kyrgyz Republic
